# Purine metabolism in lung adenocarcinoma: A single‐cell analysis revealing prognostic and immunotherapeutic insights

**DOI:** 10.1111/jcmm.18284

**Published:** 2024-04-10

**Authors:** Pengpeng Zhang, Shengbin Pei, Guangyao Zhou, Mengzhe Zhang, Lianmin Zhang, Zhenfa Zhang

**Affiliations:** ^1^ Key Laboratory of Cancer Prevention and Therapy, Tianjin's Clinical Research Center for Cancer, Department of Lung Cancer, Tianjin Lung Cancer Center, National Clinical Research Center for Cancer Tianjin Medical University Cancer Institute and Hospital Tianjin China; ^2^ Department of Thoracic Surgery The First Affiliated Hospital of Nanjing Medical University Nanjing China; ^3^ Department of Breast Surgical Oncology, National Cancer Center/National Clinical Research Center for Cancer/Cancer Hospital Chinese Academy of Medical Sciences and Peking Union Medical College Beijing China

**Keywords:** immunotherapy, LUAD, prognosis, purine metabolism, signature

## Abstract

Lung adenocarcinoma (LUAD) is a prevalent subtype of lung cancer, yet the contribution of purine metabolism (PM) to its pathogenesis remains poorly elucidated. PM, a critical component of intracellular nucleotide synthesis and energy metabolism, is hypothesized to exert a significant influence on LUAD development. Herein, we employed single‐cell analysis to investigate the role of PM within the tumour microenvironment (TME) of LUAD. PM scoring (PMS) across distinct cell types was determined using AUCell, UCell, singscore and AddModuleScore algorithms. Subsequently, we explored communication networks among cells within high‐ and low‐PMS groups, establishing a robust PM‐associated signature (PAS) utilizing a comprehensive dataset comprising LUAD samples from TCGA and five GEO datasets. Our findings revealed that the high‐PMS group exhibited intensified cell interactions, while the PAS, constructed using PM‐related genes, demonstrated precise prognostic predictive capability. Notably, analysis across the TCGA dataset and five GEO datasets indicated that low‐PAS patients exhibited a superior prognosis. Furthermore, the low‐PAS group displayed increased immune cell infiltration and elevated CD8A expression, coupled with reduced PD‐L1 expression. Moreover, data from eight publicly available immunotherapy cohorts suggested enhanced immunotherapy outcomes in the low‐PAS group. These results underscore a close association between PAS and tumour immunity, offering predictive insights into genomic alterations, chemotherapy drug sensitivity and immunotherapy responses in LUAD. The newly established PAS holds promise as a valuable tool for selecting LUAD populations likely to benefit from future clinical stratification efforts.

## 1 INTRODUCTION

Lung cancer (LC) is the second most common malignant tumour worldwide, ranking below breast cancer and its incidence is projected to reach approximately 2.2 million in 2020. Furthermore, LC remains the primary cause of cancer‐related mortality, contributing to approximately 18% of cancer deaths.^1^, ^2^ Lung adenocarcinoma (LUAD) is the most significant LC and current conventional treatments encompass surgical intervention, radiotherapy, chemotherapy and targeted therapy.^3^ Recently, the advent of immunotherapy has introduced a novel therapeutic approach for advanced LUAD patients.^4^ However, the lack of effective prognostic indicators hinders the prediction of the prognosis and response to immunotherapy of LUAD patients.

Purines represent a highly abundant group of metabolic substrates serving as crucial precursors for DNA and RNA synthesis in all cellular entities.^5^ Moreover, they play essential roles in immune system regulation and energy carrier production, providing vital support for numerous biochemical reactions. Purine metabolism (PM) actively maintains the cellular reservoir of guanosine and adenosine, constituting a pivotal component of cellular metabolic processes. This intricate pathway is meticulously regulated by an array of enzymes, so dysfunctions within these enzymes result in excessive cell proliferation and immune imbalances, thereby contributing to tumorigenesis. Previous studies have observed elevated purine metabolites in tumour cells, prompting the development of purine antimetabolite antitumor drugs which exert their effects through various mechanisms, including direct toxicity, interference with the tumour microenvironment (TME), inhibition of DNA synthesis, and disruption of DNA damage repair. Prominent classes of such drugs include 6‐Mercaptopurine, Thioguanine, Fludarabine and Cladribine, which are used to treat diverse cancers including leukaemia, lymphoma and solid tumours. Additionally, PM‐related genes (PMGs) correlate with tumour prognosis. Su et al. identified several PMGs, namely PPAT, DCK, ATIC, IMPDH1 and RRM2, as prognostic biomarkers for hepatocellular carcinoma.^6^ Similarly, Chen et al. developed a prognostic model for glioma patients based on 11 PMGs, which exhibited favourable performance in predicting patient outcomes. Furthermore, this model demonstrated potential in predicting immune cell infiltration and the expression levels of immunotherapeutic targets in glioma.^7^ Additionally, inhibiting purine synthesis in breast cancer leads to an increased pyrimidine‐to‐purine ratio, upregulation of immunoproteasome expression, and an enhanced response to immune checkpoint inhibitors.^8^

Single‐cell RNA sequencing (scRNA‐seq) is an advanced genomics technology that enables the comprehensive analysis of gene expression and genomic characteristics at the individual cell level, thereby facilitating in‐depth investigations of cellular properties. This technique offers the ability to identify and classify distinct cell types, including both tumour and normal cells, thereby supporting comprehensive cell characterization. Moreover, scRNA‐seq allows for the monitoring of gene expression dynamics during cellular development, shedding light on the underlying mechanisms governing cell differentiation and maturation. Additionally, it enables the identification of diverse cell subpopulations within tumours, playing a pivotal role in elucidating the diversity and functions of immune cells, as well as their anti‐tumour mechanisms. Notably, scRNA‐seq has been extensively employed to evaluate the prognostic implications of various tumours, encompassing lung, liver, pancreatic, breast, glioma, ovarian and gastric cancers.

In this study, scRNA‐seq data were employed to investigate the role of PMGs within the TME. Utilizing various scoring methodologies, it was observed that epithelial cells exhibited the highest purine metabolism scoring (PMS). Subsequent analyses of intercellular interactions revealed that the high PMS group displayed more robust intercellular communication. Ultimately, a robust purine metabolism‐associated signature (PAS) score was developed, incorporating data from multiple datasets. This score effectively distinguishes between cold and hot tumours within the TME, offering prognostic predictions for patients and valuable insights into the outcomes of immunotherapy treatments.

## 2 METHOD

### 2.1 Dataset Source

RNA sequencing, methylation, CNV, mutation data and clinical details for LUAD patients were sourced from the TCGA database (https://portal.gdc.cancer.gov). Additionally, the scRNA‐seq dataset GSE189357^9^ was obtained from the Gene Expression Omnibus (GEO) database (http://www.ncbi.nlm.nih.gov/geo). This dataset included samples from nine treatment‐naïve patients with LUAD, designated as TD1 through TD9, collected from patients diagnosed with various stages of the disease: adenocarcinoma in situ (AIS), minimally invasive adenocarcinoma (MIA) and invasive adenocarcinoma (IAC), providing a comprehensive overview of the disease progression. Furthermore, five additional datasets related to LUAD, all containing clinical survival information, were sourced from the GEO database for model validation, including GSE13213^10^ (*n* = 119), GSE26939^11^ (*n* = 115), GSE29016^12^ (*n* = 39), GSE30219^13^ (*n* = 86) and GSE42127^14^ (*n* = 133).

Eight datasets pertaining to immunotherapy were also collated from the GEO database, encompassing transcriptomic data and treatment response information from patients including GSE35640 (65 melanoma patients who participated in an immunological adjuvant trial using the recombinant MAGE‐A3 antigen^15^), iMvigor210 (310 bladder cancer patients treated with Atezolizumab^16^), GSE135222 (27 advanced non‐small cell LC patients were treated with anti‐PD‐1/PD‐L1 therapy^17^), GSE100797 (27 stage IV melanoma patients were treated with adoptive T‐cell therapy^18^), GSE126044 (16 non‐small cell LC patients received PD‐1 therapy^19^), GSE165252 (77 oesophageal adenocarcinoma patients were treated with neoadjuvant chemoradiotherapy combined with Atezolizumab^20^), GSE173839 (105 breast cancer patients received Durvalumab in combination with olaparib and paclitaxel^21^) and GSE103668 (21 samples from triple‐negative breast cancer patients were treated with cisplatin and bevacizumab^22^). Protein‐level data for LUAD were downloaded from the Clinical Proteomic Tumour Analysis Consortium (CPTAC, https://proteomics.cancer.gov/programs/cptac) website and subjected to logarithmic transformation (log2‐transformed) and median‐centering.

These data resources were utilized to enable a comprehensive understanding of the molecular characteristics of LUAD patients and their responses to immunotherapy. Gene‐expression data were transformed into Transcripts Per Million format, and potential batch effects were mitigated using the ‘combat’ function within the ‘sva’ R package^23^ to ensure data uniformity and comparability. Additionally, all TCGA data, including large‐scale sequencing data obtained from GEO, underwent a logarithmic transformation for standardization before analysis.

### 2.2 Cancer cell lines

Human cancer cell lines (CCLs) expression profile data were sourced from the Cancer Cell Line Encyclopedia (CCLE) project hosted by the Broad Institute (accessible at https://portals.broadinstitute.org/ccle/). Furthermore, comprehensive genome‐wide CRISPR knockout screening data for 739 cell lines, covering 18,333 genes, were acquired from the Dependency Map (DepMap) portal (available at https://depmap.org/portal/). To evaluate the reliance of specific genes on the growth and survival of CCLs, CERES scores were employed, with lower scores denoting higher gene significance within the respective CCL. Drug sensitivity data for CCLs were compiled from the Cancer Therapeutics Response Portal (CTRP) and the PRISM Repurposing dataset. The CTRP provides sensitivity metrics for 481 compounds tested on 835 CCLs, whereas PRISM offers data for 1448 compounds across 482 CCLs. Both datasets utilize Area Under the Curve (AUC) values to gauge drug sensitivity, with lower AUC values suggesting a higher treatment response. Compounds with over 20% missing data were excluded. Molecular data from the CCLE project were employed, as the CCLs in these datasets were sourced from CCLE, for an in‐depth analysis of CTRP and PRISM data.

### 2.3 The single‐cell analysis process

The raw gene expression matrix was processed using the ‘Seurat’ R package (version 4.2.0)^24^ and the genes included were required to be expressed in at least 10 cells within each sample. Subsequently, low‐quality cells were filtered out based on the following criteria: cells with more than 6000 or fewer than 200 expressed genes, or cells with over 10% unique molecular identifiers (UMIs) originating from the mitochondrial genome. The remaining high‐quality cell single‐cell transcriptome expression matrix was integrated using the ‘harmony’ R package.^25^ Then, highly variable genes were selected for principal component analysis (PCA), and the top 30 significant principal components (PCs) were chosen for t‐distributed Stochastic Neighbour Embedding (t‐SNE) dimension reduction, as well as visualization of gene expression. Differentially expressed genes (DEGs) in each cell subpopulation were identified utilizing the ‘FindAllMarker’ function, and cell types and subtypes were annotated based on the expression of well‐established canonical marker genes for each cell type. The PMS for each cell were calculated using multiple scoring methods, including AUCell, UCell, singscore and AddModuleScore, with the average of these scores representing the cell PM activity.

### 2.4 Cell–cell interactions

CellChat^26^ was used to integrate gene expression data and assess differences in the hypothesized cell–cell communication modules. The default CellChatDB was employed as the ligand‐receptor database following the standard CellChat pipeline. Cell type‐specific interactions were inferred by identifying overexpressed ligands or receptors within a cell group, followed by the identification of enhanced ligand‐receptor interactions when ligands or receptors were overexpressed.

### 2.5 The selection of key genes

The ‘findMarker’ function was employed to identify genes exhibiting notable differences between groups with high‐ and low‐PMS. Genes demonstrating a fold change (FC) >1.5 were deemed significant and then, Spearman correlation analysis was conducted to pinpoint the top 150 genes displaying the strongest correlation with the PMS. These highly correlated genes were then amalgamated with the significant DEGs, forming the basis for the development of subsequent predictive models.

### 2.6 Building the high‐performance PAS

Univariate Cox regression was used to analyse the effect of key genes on LUAD survival (*p* > 0.05). Least Absolute Shrinkage and Selection Operator (LASSO) Cox regression then narrowed down these genes to form an optimal survival signature. The model's effectiveness was evaluated by ROC curves, with an AUC over 0.65 indicating good performance.

### 2.7 Mutation landscape

Genomic alterations, including recurrently amplified and deleted regions, were identified through GISTIC 2.0 analysis. The R package ‘maftools’^27^ was utilized to calculate the tumour mutation burden (TMB).

### 2.8 Differences in the TME and drug inference

Seven distinct immune infiltration algorithms were employed to assess the composition of immune cells across various PAS groups. These algorithms enabled a detailed analysis of the nuanced differences in immune cell infiltration. Heatmaps were used to visually represent these variances, highlighting the subtle disparities in immune cell populations. Furthermore, the advanced functionalities of the ‘estimate’ R package^28^ were utilized to calculate immune scores, stromal scores, and ESTIMATE scores for patients in the TCGA‐LUAD database. This approach significantly enriched the comprehensive evaluation of the TME and its potential impacts on patient outcomes. The predictive power of the ‘oncoPredict’ and ‘pRRophetic’ R packages^29^, ^30^ was applied to identify effective chemotherapeutic agents for different risk groups. This application facilitated the prediction of suitable therapeutic approaches for more informed and strategic treatment decisions.

### 2.9 GSVA enrichment analysis

Gene set enrichment analysis was performed leveraging 50 hallmark pathways from the Molecular Signatures Database (MSigDB). Pathway activity estimates for each cell type were derived using gene set variation analysis (GSVA) for individual cells, with subsequent averaging of gene expression levels for each cell subtype employing default parameters in the GSVA package.^31^ Differential pathway activity across diverse cell subtypes was quantified based on variations in activity scores.

### 2.10 Clinical specimen collection and RNA sequencing

Ethical approval was granted by the Medical Ethics Committee of the First Affiliated Hospital of Nanjing Medical University for the collection of tissue samples. These samples, which have been assessed by pathology experts as AIS, MIA or IAC, were collected on the day of the surgery and subsequently transported to Oncocare Inc. (Suzhou, China) for RNA sequencing.

### 2.11 Immunohistochemistry

Paraffin‐embedded tissue sections were incubated for 120 min at 37°C with the primary antibodies anti‐CD8A (1:2000 dilution; Cat#ab217344; Abcam, USA) and PD‐L1 (1:5000 dilution; Cat#66248‐1‐Ig; Proteintech, Wuhan, China). Following this, HRP‐conjugated secondary antibodies were applied and incubated for 30 minutes at the same temperature. The sections were then stained with DAB (3,3′‐diaminobenzidine) and counterstained with haematoxylin^32^ for visualization.

### 2.12 Statistical analysis

All data processing, statistical analysis and visualization tasks were conducted using R software, version 4.2.0. the Kaplan–Meier method and the log‐rank test were employed to estimate and compare subtype‐specific overall survival. Differences in continuous variables between groups were evaluated using either the Wilcoxon test or the *t*‐test depending on the data distribution. Categorical variables were analysed using either the chi‐squared test or Fisher's exact test. The FDR method for *p*‐value correction was applied to adjust for potential false discovery rates. Correlations between various variables were determined using Spearman correlation analysis. All *p*‐values were computed using a two‐tailed approach and the threshold for statistical significance was *p* < 0.05.

## 3 RESULTS

### 3.1 A scRNA‐seq approach to cellular heterogeneity and purine metabolism dynamics

**FIGURE 1:**
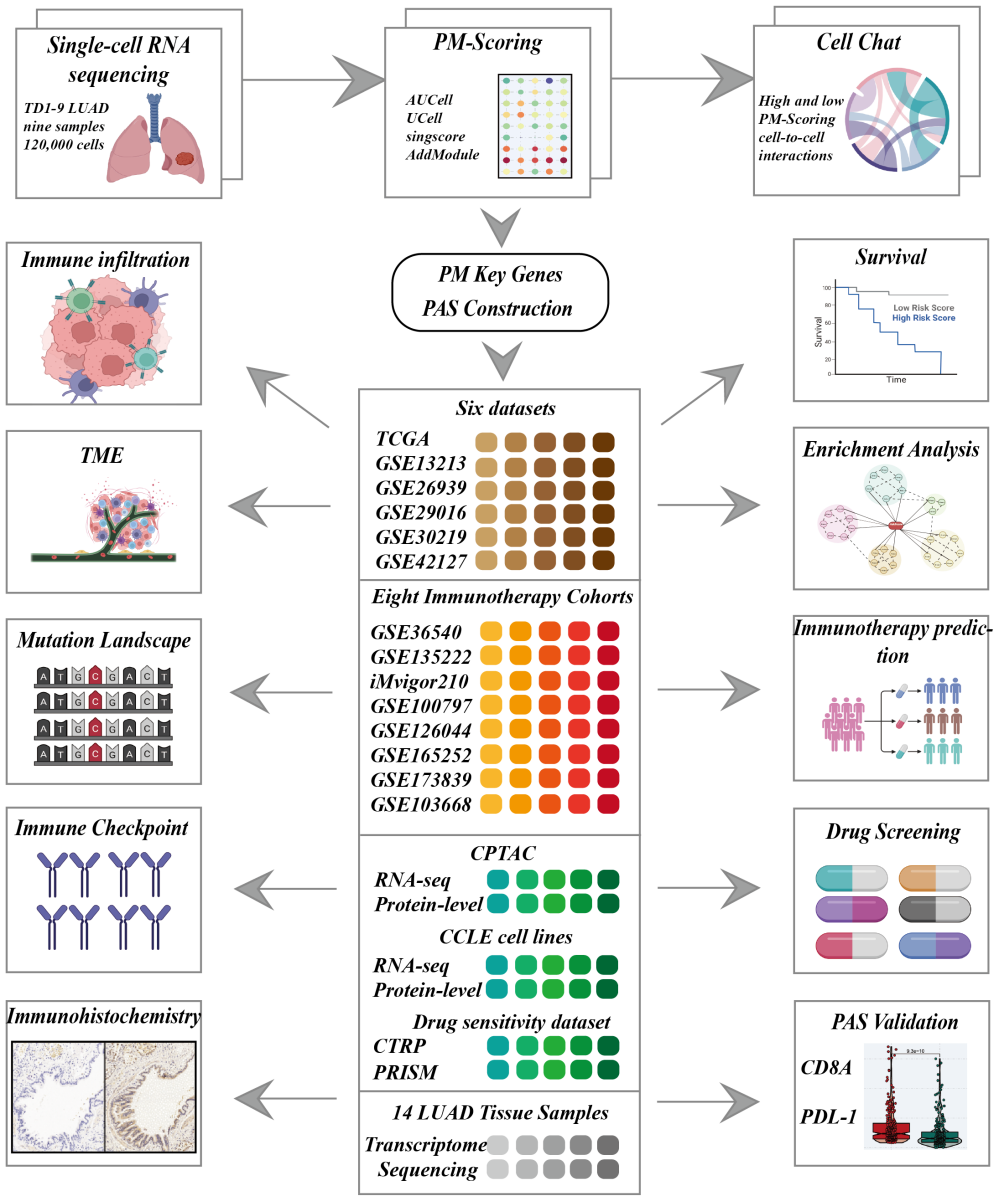
Study methodology flowchart.

**FIGURE 2:**
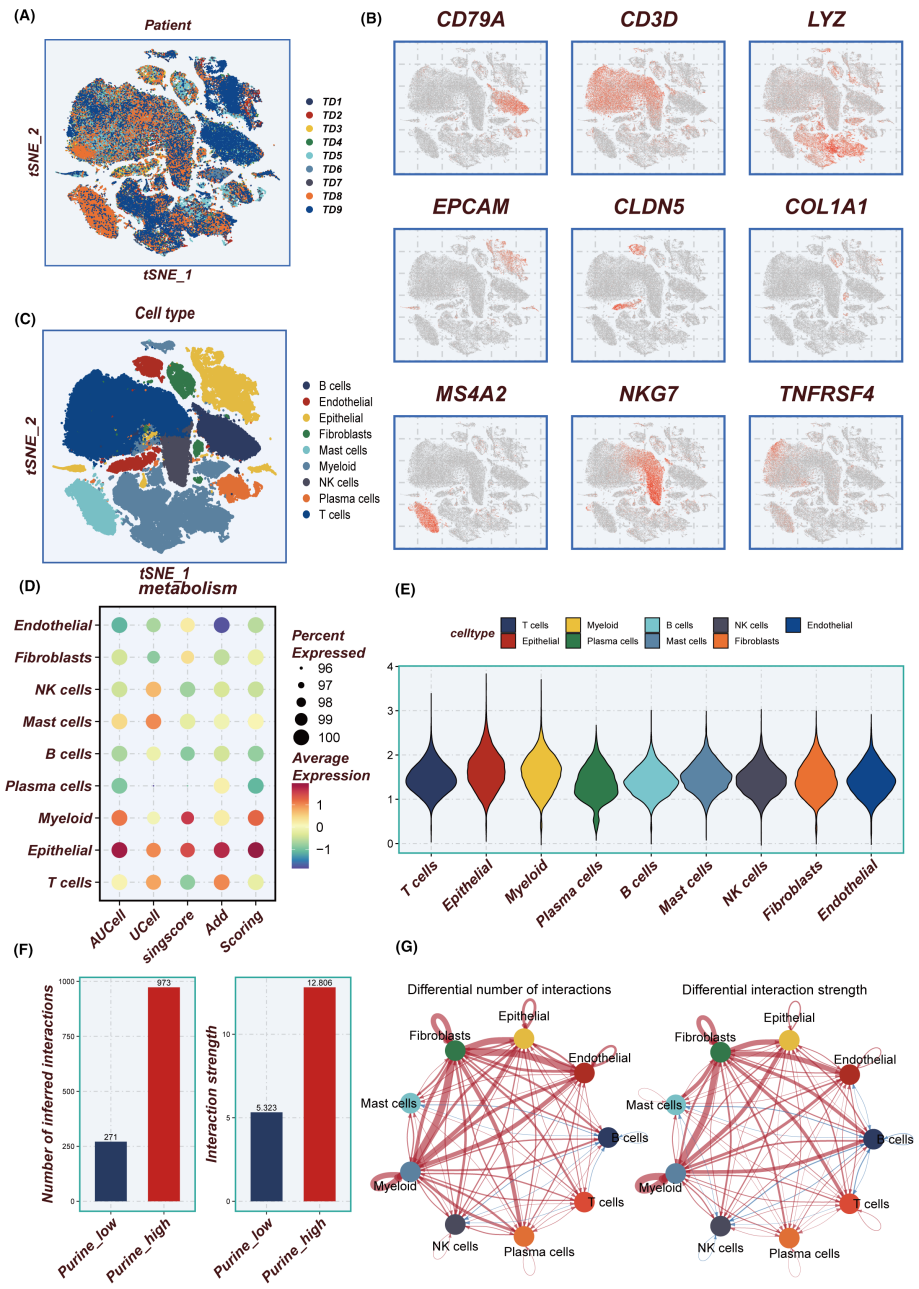
Cell types annotation and cell communication. (A) Sample distribution without significant batch effects. (B) Marker genes for typical cell types. (C) tSNE plot demonstrating dimensionality reduction clustering. (D) Bubble plots showing four methods to assess the average expression level of PMGs in each cell type. (E) Violin plot showing scoring of each cell type. (F, G) Differences in the number and intensity of cellular communication between high‐ and low‐ PMS groups.

Figure 1 depicts the flowchart outlining the study methodology. Initially, the scRNA‐seq data underwent rigorous quality control measures and was subjected to dimensionality reduction. A total of nine samples were included in this investigation, and the distribution of cells within each sample is presented in Figure 2A. Each cluster was identified as a specific cell subtype, such as epithelial cells, endothelial cells, fibroblasts, myeloid cells, B cells, plasma cells, NK cells, T cells and macrophages, based on the expression of typical markers and most DEGs (Figure 2B,C). The average expression levels of PMS across different cell types were assessed using four distinct algorithms (Figure 2D), with epithelial cells and myeloid cells in the TME exhibiting the highest PMS (Figure 2E).

### 3.2 Cell–cell interactions

As shown in Figure 2F, a significantly greater number, and strength of intercellular communication were exhibited by the high‐PMS group compared to the low‐PMS group, particularly in fibroblasts, epithelial, myeloid and endothelial cells (Figure 2G). The number and proportion of different signalling pathways between the high‐PMS and low‐PMS groups are depicted in Figure S1A,B. Furthermore, the expression profiles of ligand‐receptor pairs in different cell populations varied between the distinct PMS groups, as demonstrated in Figure S1C,D. The analysis of the ligand‐receptor pairs depicted distinctly highlights an enhanced interaction between collagen molecules and other mediators such as integrin (ITGA3/ITGB1) and glycoprotein (CD44) within the high PM subgroup. This upregulation of molecular interplay, particularly in a purine‐rich metabolic milieu, suggests a pivotal role in cancer progression. The increased collagen‐integrin interaction may facilitate the remodelling of the extracellular matrix, thus promoting tumour invasiveness and metastasis. Simultaneously, the elevated engagement of CD44 points to a potential mechanism by which cancer cells may enhance their adhesive properties, favouring dissemination. These interactions underline the complex network of cell communication that may contribute to the dynamic TME, offering potential targets for therapeutic intervention in the oncogenic process. It is worth noting that in Figure S1E, a significant upregulation in both signal transmitting intensity and signal receiving intensity was observed across all cell types in the high‐PMS group compared to the low‐PMS group.

### 3.3 Identification of key purine metabolism genes and prognostic signature

**FIGURE 3:**
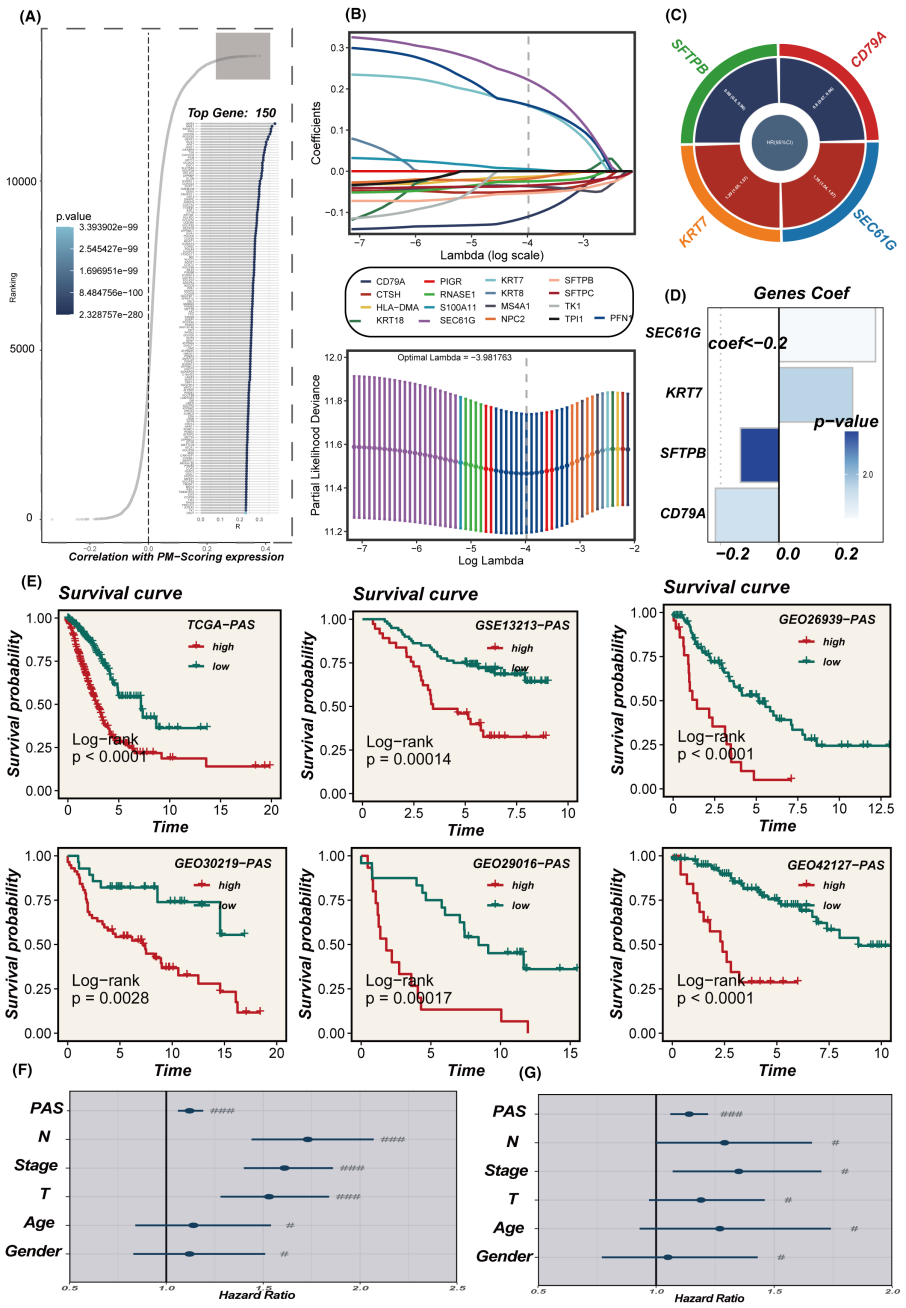
Model development and survival analysis. (A) The top 150 genes with the highest PMS correlations. (B) LASSO regression analysis of the TCGA cohort. (C) Circle plot showing results of multivariate Cox regression analysis. (D) Distribution of modelled gene coefficient values. (E) Survival curves of the TCGA and GEO cohorts. (F, G) Univariate and multivariate Cox regression analysis of clinical variables and PAS scores in the TCGA cohort. ###means *p* < 0.01; ##means *p* < 0.05; #means *p* > 0.05.

The identification of the top 150 pivotal PM genes was achieved via Spearman correlation analysis for model development (Figure 3A). This process entailed integrating DEGs in the high‐ and low‐PMS groups using the ‘findmarker’ function (*p* < 0.05, FC >1.5) with the top 150 correlated genes (correlation >0.2, *p* < 0.05). Univariate Cox regression analysis was performed on this integrated data set and the subsequent construction of the PAS involved meticulous Lasso and COX regression analyses (Figure 3B). The HR values, their associated confidence intervals, and the distribution of coefficients employed in the model construction using the four pivotal genes are presented in Figure 3C,D. Notably, within these model genes, SFTPB and CD79A emerged as protective factors (HR <1), whereas KRT7 and SEC61G were identified as risk factors (HR >1).

### 3.4 Stratification of patients and validation of the PAS

**FIGURE 4:**
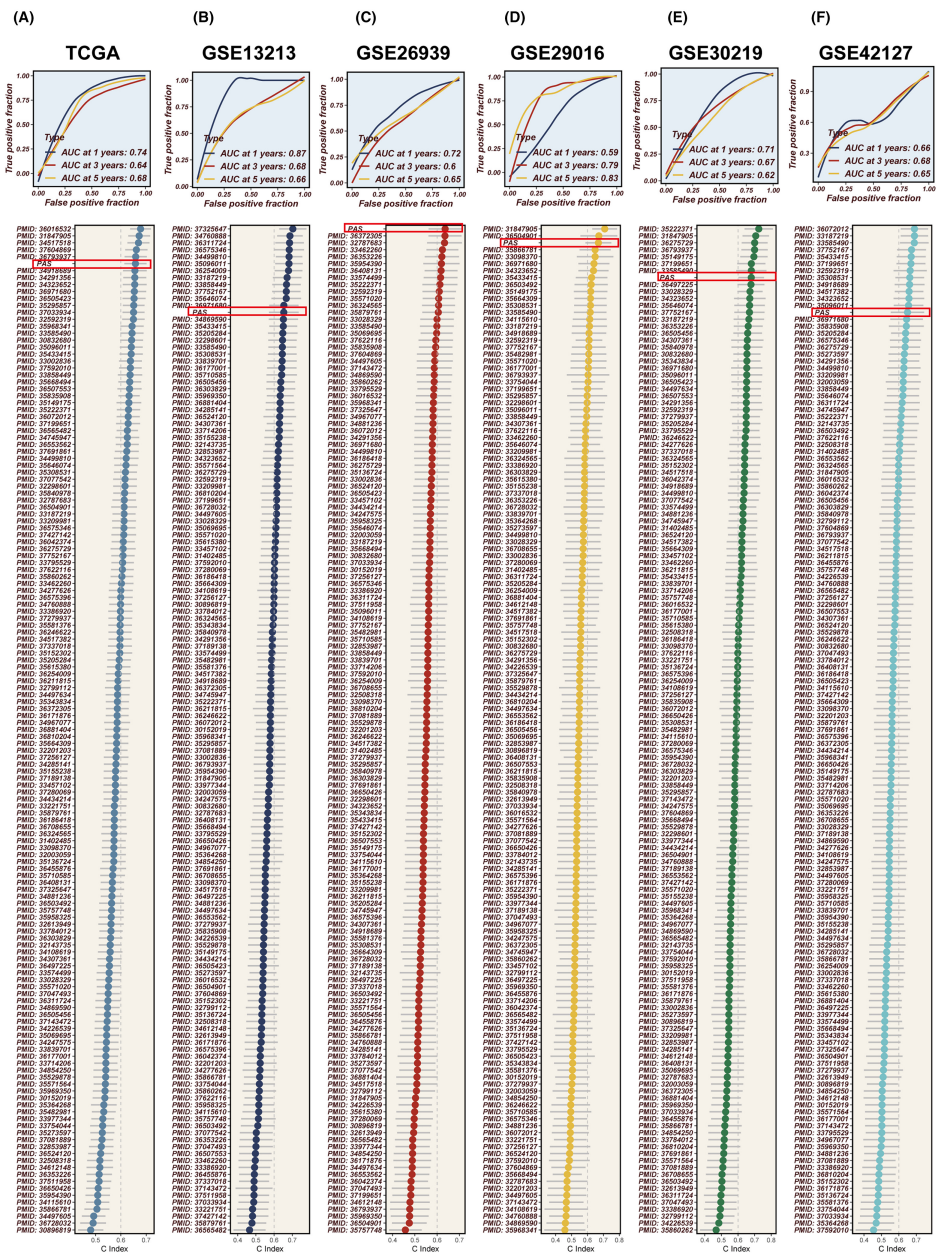
Model evaluation in six datasets. (A–F) Time‐dependent ROC curves of the PAS regarding 1‐,3‐ and 5‐year OS and the C‐index comparing of the PAS and 144 LUAD models in the TCGA, GSE13213, GSE26939, GSE29016, GSE30219 and GSE42127 datasets.

All patients were classified into two distinct groups, the high‐PAS group and the low‐PAS group, based on the constructed PAS of the median PAS scores. As demonstrated in Figure 3E, patients belonging to the high‐PAS group exhibited significantly worse prognoses compared to those in the low‐PAS group within the TCGA dataset. This pattern was confirmed in the GSE13213, GSE26939, GSE29016, GSE30219 and GSE42127 cohorts. PCA analysis was conducted to assess the separation of sample populations between the high‐ and the low‐PAS groups, demonstrating effective segregation of the two groups into distinct clusters, thereby highlighting the model accuracy and stability (Figure S2). Univariate and multivariate COX regression analyses determined that the PAS scores derived from the model could serve as an independent prognostic determinant, augmenting the predictive power for patient outcomes in LUAD. (Figure 3F, G). The performance of the model in predicting 1, 3 and 5‐year survival in LUAD patients was evaluated using ROC curves (Figure 4A–E), with the model being highly accurate in predicting survival outcomes across multiple datasets. Subsequently, a collection of 144 previously published signatures from the medical literature was compared to our constructed PAS, revealing that the c‐index values of our PAS consistently ranked prominently, with top‐ten rankings observed in TCGA, GSE26939, GSE29016 and GSE30219. This underscores the exceptional precision of the PAS in the context of medical research.

### 3.5 Heatmap analysis and nomogram construction for enhanced prognostic assessment

The visualization of the distribution of each clinical feature and model gene in different subgroups was achieved through a heatmap analysis. Figure S3A demonstrates a statistically significant difference between the two groups concerning T and N stage, clinical stage and fustat (*p* < 0.05). Notably, the high‐PAS group exhibited more advanced clinical staging, N stage and T stage, but a lower proportion of older patients (Figure S3B). To enhance the convenience and accuracy of prognostic prediction for LUAD patients, a nomogram was constructed integrating clinical characteristics and PAS scores (Figure S3C). This serves as a valuable tool for clinicians to more precisely assess the risk of patients and guide future treatment decisions. Subsequently, decision curves, c‐index curves and calibration curves analysis were used to evaluate the accuracy and consistency of the nomogram compared to other clinical indicators in predicting patient prognosis (Figure S3D–F), indicating that the nomogram exhibits higher accuracy and consistency, thus it is a potential clinical decision‐making tool. Moreover, a comprehensive prognostic ROC analysis (Figure S3G–I) was conducted to assess the accuracy of the nomogram with AUC values of 0.740, 0.743 and 0.750 at years 1, 3 and 5, respectively, emphasizing the reliable predictive capability of the nomogram in assessing the prognosis of LUAD patients.

### 3.6 Elucidating the relationship between the PAS score, oncogenic pathways and the cancer‐immunity cycle

**FIGURE 5:**
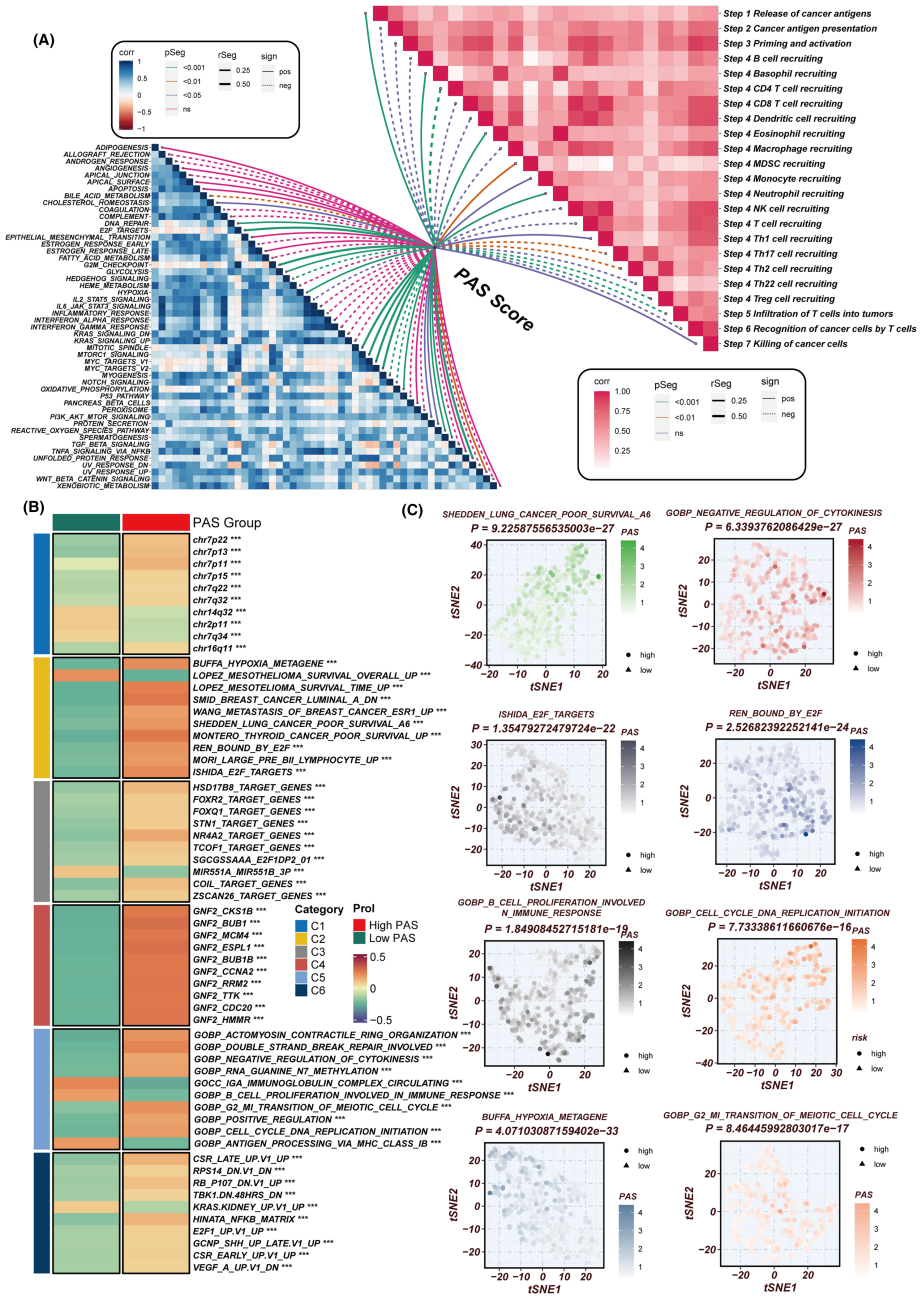
Biological properties of the PAS in TCGA dataset. (A) The correlation of PAS scores with the steps in cancer‐immunity cycle and the hallmark pathways. (B) GSVA enrichment analysis delineated the biological attributes of high‐ and low‐PAS groups. (C) tSNE plot of GO and KEGG terms delineated the differences in pathways activity in high‐ and low‐PAS groups.

The exploratory analysis into the relationship of the PAS score with established hallmark oncogenic signatures and the cancer‐immunity cycle unveiled a pronounced positive correlation with pathways that are quintessential for cell cycle progression and survival, such as DNA repair mechanisms, E2F transcription factor targets, processes involved in the epithelial‐mesenchymal transition (EMT), and the G2M checkpoint. These associations underscore the potential of the PAS score to reflect the proliferative and aggressive nature of LUAD, given that DNA repair capability and E2F target gene expression are critical for the rapid proliferation of cancer cells. Similarly, EMT is a fundamental process by which epithelial cells acquire mesenchymal, migratory, and invasive properties, facilitating metastasis. Moreover, the regulation of the G2M checkpoint is pivotal for maintaining genomic stability, and its dysregulation is often implicated in oncogenesis. Conversely, an inverse correlation was discerned between the PAS score and critical facets of the cancer‐immunity cycle, which encompasses the presentation of cancer antigens, the recruitment of cytotoxic CD8^+^ T cells, and their infiltration into the tumour parenchyma (Figure 5A). This negative correlation may indicate an immunosuppressive TME fostered by higher PAS scores, wherein the effective presentation of tumour antigens is diminished, consequently hampering the recruitment and infiltration of T cells that are instrumental for anti‐tumour immunity. The attenuation of these immune steps could be reflective of an immune evasion strategy by the tumour, thereby contributing to cancer progression and highlighting the PAS score as a potential biomarker for the immunogenicity and immunotherapy responsiveness of LUAD. Further analysis confirmed these results, as depicted in Figure 5B,C. The low‐PAS group was significantly enriched in pathways involving B cell proliferation involved in the immune response and antigen processing and presentation of endogenous lipid antigen via MHC class IB, while the high‐PAS group exhibited significant enrichment in pathways related to G2M transition of the meiotic cell cycle, Shedden LC poor survival, and cell cycle DNA replication initiation. In conclusion, our findings suggest that a low PAS score indicates a higher potential for an immune response under immunotherapy.

### 3.7 A significant correlation between the PAS score and immune infiltration characteristics

**FIGURE 6:**
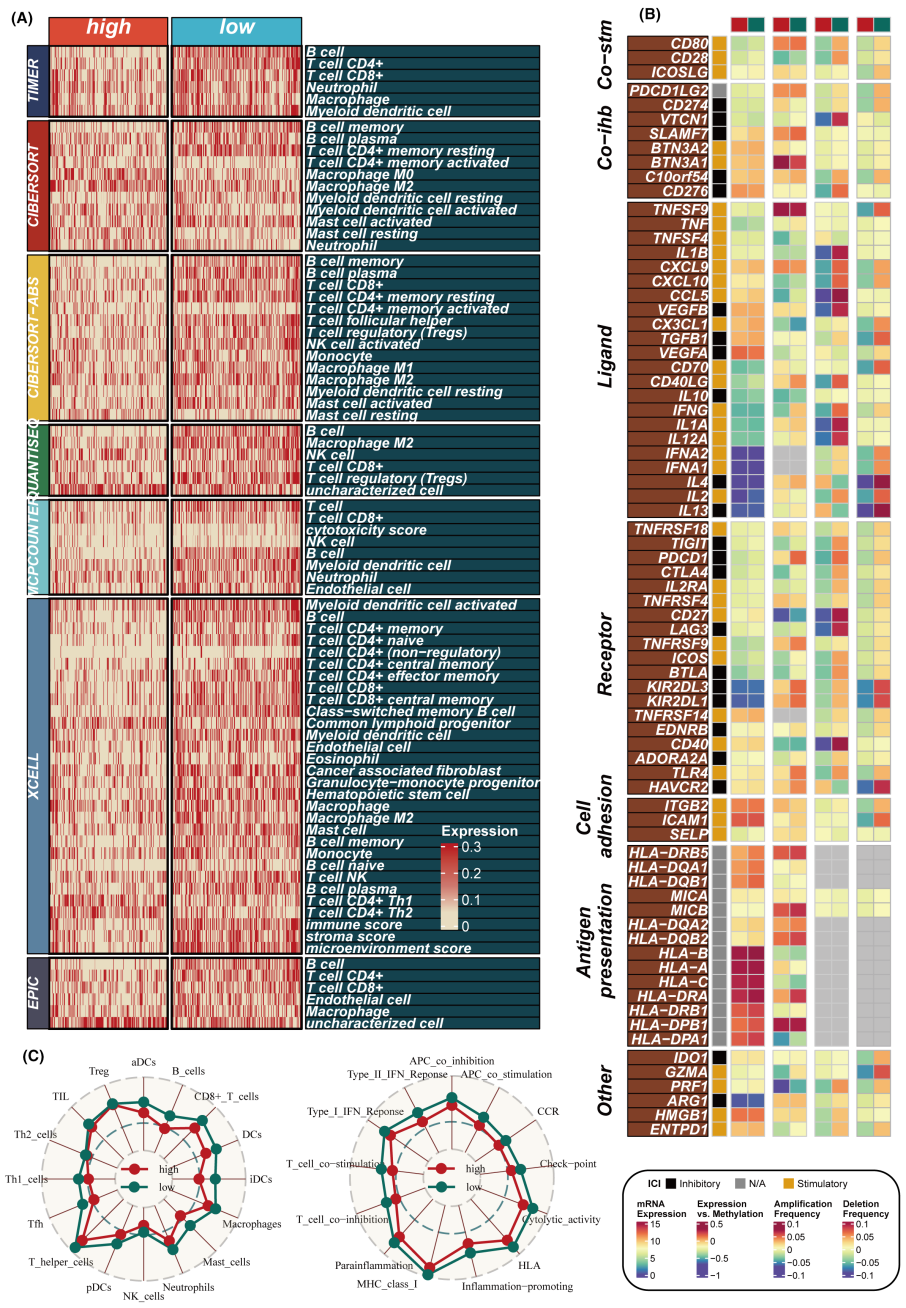
Immune infiltration assessment. (A) A heatmap shows the immune infiltration of two PAS groups. (B) The correlation between the PAS and immune modulators. (C) Assessment of immune cell infiltration and immune‐related pathways differences between two PAS groups with the ssGSEA algorithm.

To explore the immune status reflected by the PAS score, we analysed the relationship between the PAS score and immune‐infiltrating cells as well as immune checkpoints. The low‐PAS group in the TCGA dataset exhibited higher levels of immune‐infiltrating cells and immune modulators, indicating an inflammatory but relatively immune‐promoting microenvironment, suggesting potential immunotherapy benefits (Figure 6A,B). Furthermore, there were some differences in methylation levels and CNV between the high‐PAS and low‐PAS groups. The ssGSEA method was employed to evaluate the disparities in immune cell infiltration and immune‐related pathways between the high‐ and low‐PAS groups, revealing that the low‐PAS group exhibited higher levels of immune cell infiltration, including B cells, iDCs and mast cells. Furthermore, the low‐PAS group displayed greater activity in specific immune‐related pathways, such as the type II interferon (IFN) response and T‐cell co‐stimulation (Figure 6C). Additionally, the ‘estimate’ R package was utilized to assess the level of immune infiltration, and correlation analysis demonstrated a significant negative correlation between the PAS score and the immune score, along with a positive correlation with tumour purity (Figure S4). These findings highlight distinct immune characteristics and pathways associated with the high‐ and low‐PAS groups, providing insights into the underlying immune landscape and potential therapeutic targets.

### 3.8 Multiomic alterations associated with the PAS score

**FIGURE 7:**
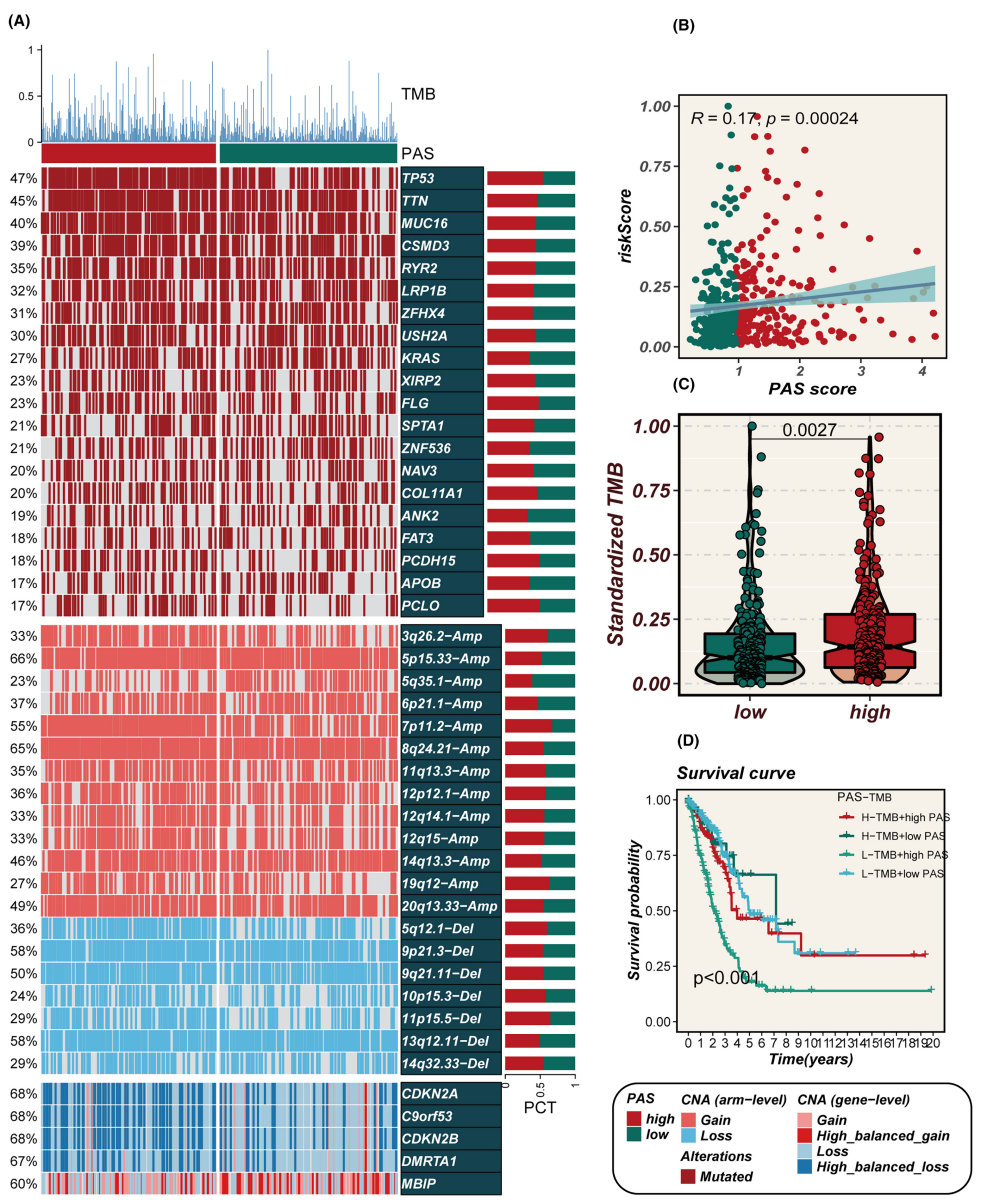
Muti‐omics alteration characteristics of PAS in TCGA dataset. (A)Genomic alteration landscape in high‐ and low‐PAS groups. (B)The correlation between the PAS and TMB. (C)The fraction of standardized TMB in high‐ and low‐PAS groups. (D)Survival curves show the difference of survival among four subgroups (high‐PAS and high‐TMB, high‐PAS and low‐TMB, low‐PAS and high‐TMB, low‐PAS and low‐TMB).

Figure 7A displays alterations in specific genomic regions between different PAS groups. Among the identified gene variants, the top five genes in terms of mutation frequency were TP53, TTN, MUC16, CSMD3 and RYR2. Additionally, it was observed that the PAS score exhibited a positive correlation with TMB (Figure 7B). The high‐PAS group displayed a significantly higher TMB compared to the low‐PAS group (*p* = 0.0027, Figure 7C). Further stratification of patients based on both PAS score and TMB demonstrated that the low‐TMB and high‐PAS groups had the poorest prognosis (Figure 7D). These findings highlight the importance of considering both PAS score and TMB as valuable prognostic indicators in assessing patient outcomes.

### 3.9 Identifying potential drugs for the treatment of high‐PAS individuals

**FIGURE 8:**
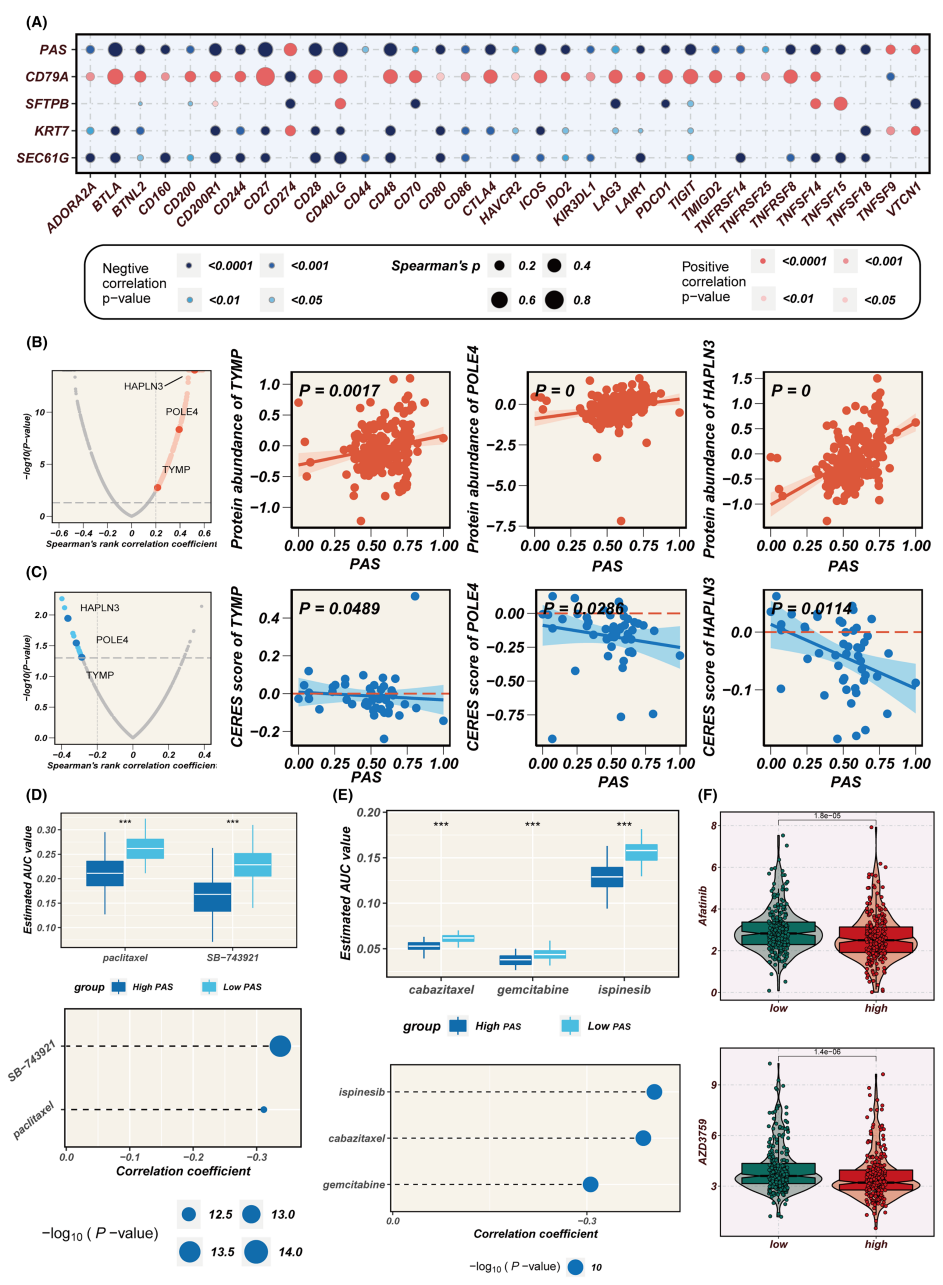
Identification of high‐PAS related drug targets and candidate agents. (A) Scatter plots demonstrate the correlation between PAS and model genes and immune checkpoint expression. (B) Volcano plot and scatter plots of spearman's correlations and significance between PAS and protein expression of drug targets. (C) Volcano plot and scatter plots of Spearman's correlations and significance between PAS and CERES score of drug targets. (D) The results of Spearman's correlation and differential drug response analysis of two CTRP‐derived compounds. (E) The results of Spearman's correlation and differential drug response analysis of three PRISM‐derived compounds. (F) The difference of drug response in two PAS groups. ***means *p* < 0.001.

Considering the pivotal role of immune checkpoints in the success of tumour immunotherapy, an investigation was conducted into the differential expression of immune checkpoint genes between the high‐ and low‐PAS groups. Correlation analysis, as depicted in Figure 8A, revealed the relationship between PAS scores, model genes and immune checkpoint gene expression. The colour scheme employed in the analysis indicated either a positive correlation (in red) or a negative correlation (in blue). The PAS score was significantly negatively correlated with the expression of several immune checkpoint genes, including BTLA, CD27, CD48, etc. Interestingly, there was a strong positive correlation between the PAS score and CD274 (PD‐L1), suggesting that the high‐PAS group might have activated potential PD‐L1‐mediated immune evasion, thus promoting tumour progression.

Proteins that exhibited a high correlation with the PAS score may be of potential therapeutic significance for patients with a high PAS score. However, most human proteins cannot be targeted pharmacologically due to a lack of well‐defined active sites for small molecule binding. Therefore, a two‐step analysis was conducted to identify candidate therapeutic targets for patients with a high PAS score and a poorer prognosis. First, the correlation between the drug‐protein expression levels and the PAS score was calculated to identify drug proteins (correlation coefficient >0.2, *p* < 0.05). Subsequently, the analysis of the correlation between CERES scores and PAS scores in LUAD cell lines identified three genes including TYMP, POLE4 and HAPLN3 (Figure 8B,C). It is of note that in most LUAD cell lines, the CERES scores for POLE4 and HAPLN3 are below zero, suggesting that inhibiting the function of these two genes in patients with a high PAS score may lead to favourable treatment outcomes, making them potential therapeutic targets.

Gene expression profiles and drug sensitivity data for LUAD cell lines were extracted from CTRP and PRISM to construct a drug response prediction model. Considering that transcriptome data from clinical samples can be influenced by normal and stromal components, the ISOpure algorithm was employed to purify tumour sample data. Subsequently, the pRophetic package was utilized to predict drug responses based on the purified LUAD tumour samples, resulting in AUC values for drug responses in each sample. Differential analyses were conducted for the high‐PAS score and low‐PAS score groups using drug response data obtained from both CTRP and PRISM. Subsequently, compounds with a negative correlation coefficient of less than 0.3 were filtered based on Spearman correlation analysis between AUC values and PAS scores, resulting in the identification of two CTRP‐derived compounds, paclitaxel and SB‐743921, as well as three PRISM‐derived compounds, cabazitaxel, gemcitabine and ispinesib (Figure 8D, E). Furthermore, the ‘oncopredict’ R package was used to explore potentially effective chemotherapeutic agents for both the high and low PAS groups, indicating that Axitinib and AZD3759 may be more effective in high‐PAS patients (Figure 8F). These results provide valuable information for tailoring treatment strategies to optimize therapy for high‐PAS LUAD patients.

### 3.10 Analysis of PAS scores in immunotherapy data sets across multiple cancers

**FIGURE 9:**
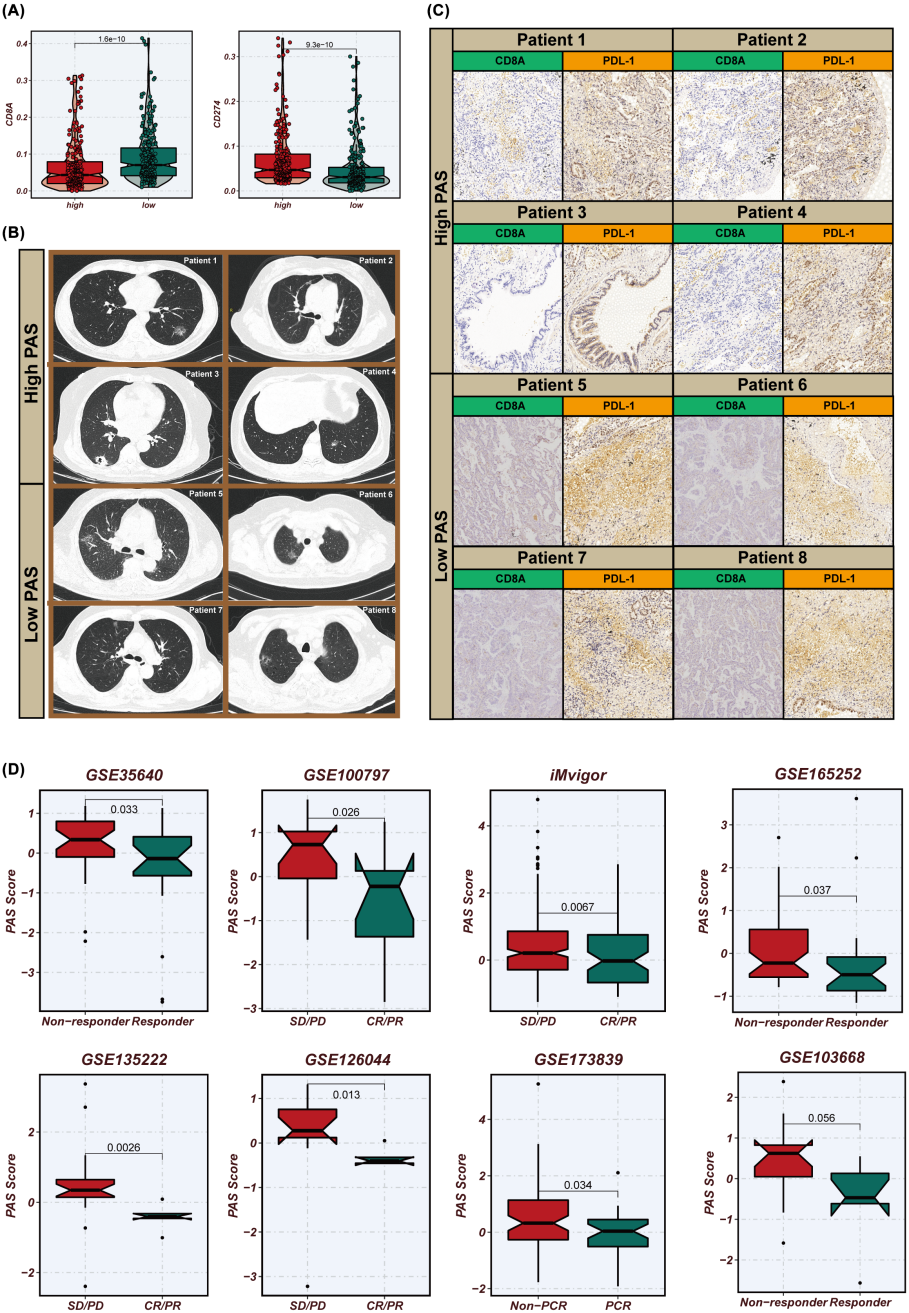
Immunotherapy datasets for prediction of PAS. (A)The different expression of CD8A and CD274 in two PAS groups. (B) Clinical samples: imaging data of high‐ and low‐PAS group patients. (C) Immunohistochemical staining images of high‐ and low‐PAS group samples clearly show higher CD8A expression and lower PD‐L1 expression in the low PAS group. (D)The association between immunotherapy response and the PAS of GSE35640, GSE100797, iMvigor210, GSE165252, GSE135222, GSE126044, GSE173839 and GSE103668 datasets.

Prior research has demonstrated that CD8A is associated with the presence of CD8^+^ T cells which can exert anti‐tumour effects, indicating better immunotherapy outcomes, whereas PD‐L1 is an immune checkpoint protein linked to tumour cells evading immune responses, potentially leading to poor patient prognosis. Our study found that the low‐PAS group exhibited higher CD8A expression, while the high‐PAS group showed higher PD‐L1 expression (Figure 9A). To validate our analysis, we collected 14 LUAD samples for transcriptome sequencing and estimated the PAS scores. The top four samples with the highest PAS scores were categorized as the high‐PAS group, and the four samples with the lowest PAS scores were classified as the low‐PAS group (Figure 9B). Immunohistochemistry confirmed that the low‐PAS group had higher CD8A expression and lower PD‐L1 expression compared to the high‐PAS group (Figure 9C). We curated eight publicly available immunotherapy datasets, encompassing melanoma, breast cancer, LC and oesophageal cancer, which included transcriptomic profiles and post‐treatment response data of patients. Utilizing the model formula, we calculated the PAS scores for these cohorts. Intriguingly, across all datasets, a pattern emerged where lower PAS scores were associated with improved responses to immunotherapy. This observation may be linked to our previous validation that demonstrated a higher CD8^+^ T cell infiltration in tumours with lower PAS scores. Such correlation suggests that the PAS score could potentially serve as a predictive biomarker for the efficacy of immunotherapy across different cancer types, reflecting the immunological landscape and its influence on treatment outcomes (Figure 9D).

## 4 DISCUSSION

LC represents a significant global public health concern, imposing a substantial healthcare burden amounting to trillions of dollars annually worldwide.^1^ Despite this, the efficacy of current LC treatments remains unsatisfactory, posing a grave threat to patients' right to life and well‐being. In this context, immunotherapy has emerged as a novel and promising approach for LUAD. However, the complex and uncertain nature of immunotherapy results in a lack of benefit for many patients, coupled with the occurrence of severe adverse effects. Consequently, the development of robust predictive tools becomes imperative to assess LUAD patient prognosis.^33^ PM, a fundamental metabolic process implicated in tumour growth, has a critical role in cancer progression. Notably, PMGs hold the potential to serve as prognostic indicators for LUAD.^7^

In this study, single‐cell analytics were employed to elucidate the potential role of PMGs within the TME. We observed that the high PMS group exhibited elevated levels of intercellular interaction, including the potential activation of collagen molecules and other receptors, which may contribute to poor prognoses. Through further analysis, we pinpointed the most critical PM genes for subsequent model validation. The Lasso‐Cox analysis was instrumental in refining the PAS score, which consistently demonstrated superior prognostic value and performance across six independent datasets.

Furthermore, a search of 144 previously published articles related to LUAD signatures revealed that the developed PAS exhibited superior performance, ranking among the top in almost all datasets. Further analysis revealed that tumour samples with low PAS scores had more immune cell infiltration and lower PD‐L1 expression. This was confirmed through multiple immune therapy datasets, with low‐PAS patients exhibiting better responses to immune therapy. To support this hypothesis, clinical samples were collected for validation, revealing that low‐PAS samples had higher CD8A staining intensity and lower PD‐L1 staining intensity. This further emphasized that the developed PAS score has the potential to serve as a clinical classifier for LUAD patients.

Nonetheless, this study has several limitations. First, while the PAS model and its scoring system were developed, further investigations are essential to gain a deeper understanding of the specific biological functions and mechanisms associated with the PAS model genes. Their roles and interactions must be validated through in‐depth in vitro and in vivo experiments. Second, the validation of the PAS score as a prognostic tool should have been extended by multi‐center cohorts. Collaborating with multiple institutions could have provided a more diverse and comprehensive dataset, thus strengthening the reliability and generalizability of our findings. Lastly, the full potential of the PAS score in predicting immune therapy responses requires further examination with the inclusion of additional LUAD immune therapy datasets. Expanding the analysis to include a larger pool of immune therapy data could enhance the robustness of this predictive tool.

In summary, the PAS score developed based on a thorough analysis of bulk RNA‐seq and scRNA‐seq data for LUAD effectively distinguished LUAD patient prognosis and predicted the efficacy of immune therapy. As a newly discovered predictive biomarker, the PAS score demonstrated the potential to accurately identify LUAD patients who would benefit from immune therapy, however, further rigorous validation is required for clinical application.

## AUTHOR CONTRIBUTIONS

**Pengpeng Zhang:** Conceptualization (equal); data curation (equal); software (equal). **Shengbin Pei:** Data curation (equal); investigation (equal); writing – original draft (equal). **Guangyao Zhou:** Data curation (equal); supervision (equal). **Mengzhe Zhang:** Data curation (equal); validation (equal). **Lianmin Zhang:** Data curation (equal); supervision (equal). **Zhenfa Zhang:** Investigation (equal); visualization (equal); writing – review and editing (equal).

## FUNDING INFORMATION

This work was supported by the Tianjin Natural Science Foundation under Grant/Award Number: 21JCYBJC01020.

## CONFLICT OF INTEREST STATEMENT

It is hereby declared by the authors that the research was carried out without the presence of any potential conflict of interest arising from commercial or financial relationships.

## CONSENT TO PARTICIPATE

Prior to their participation, all subjects provided their informed consent for inclusion.

## Supporting information


**Figure S1.** Cell–cell interaction analysis. (A, B) Differential cell–cell interaction pathways between high and low PMS groups, illustrating the variance in intercellular communication mechanisms attributed to purine metabolism activity. (C, D) Variability in ligand‐receptor pairs facilitating cell–cell interactions across high and low PMS groups, highlighting the distinct molecular dialogue engaged by varying purine metabolic states. (E) Comparative analysis of communication strength among different cell types within high and low PMS groups, revealing alterations in intercellular signalling intensity associated with purine metabolic levels.

**Figure S2.** PCA analysis shows that PAS scores can separate samples effectively in the TCGA, GSE13213, GSE26939, GSE29016, GSE30219 and GSE42127 datasets.

**Figure S3.** Clinical correlation analysis. (A)Heatmap constructed by combining clinical features and model gene expression demonstrates the distribution of clinical features and model genes in two PAS groups. (B) Differences between high‐ and low‐PAS expression groups in terms of age, T, N and clinical stage. (C) Column line graph presenting a prognostic model for LUAD patients. (D) Decision curves. (E) C‐index curves. (F) Calibration curves. (G–I) ROC curves reflected the accuracy of the NOMO score in predicting prognosis. ***means *p* < 0.001; **means *p* < 0.01; ***means *p* < 0.05.

**Figure S4.** The ESTIMATE R package was used to predict the correlation between PAS scores and immune scores, stromal scores, ESTIMATE scores and tumour purity.

## Data Availability

The datasets analysed in the current study are available in the TCGA repository (http://cancergenome.nih.gov/), and GEO (https://www. ncbi.nlm.nih.gov/geo/).
