# Supplementary figures and images for: Purine metabolism in lung adenocarcinoma: A single‐cell analysis revealing prognostic and immunotherapeutic insights
Source: J Cell Mol Med. 2024 Apr 10;28(8):e18284. doi: 10.1111/jcmm.18284 (PMC11005461; doi:10.1111/jcmm.18284)

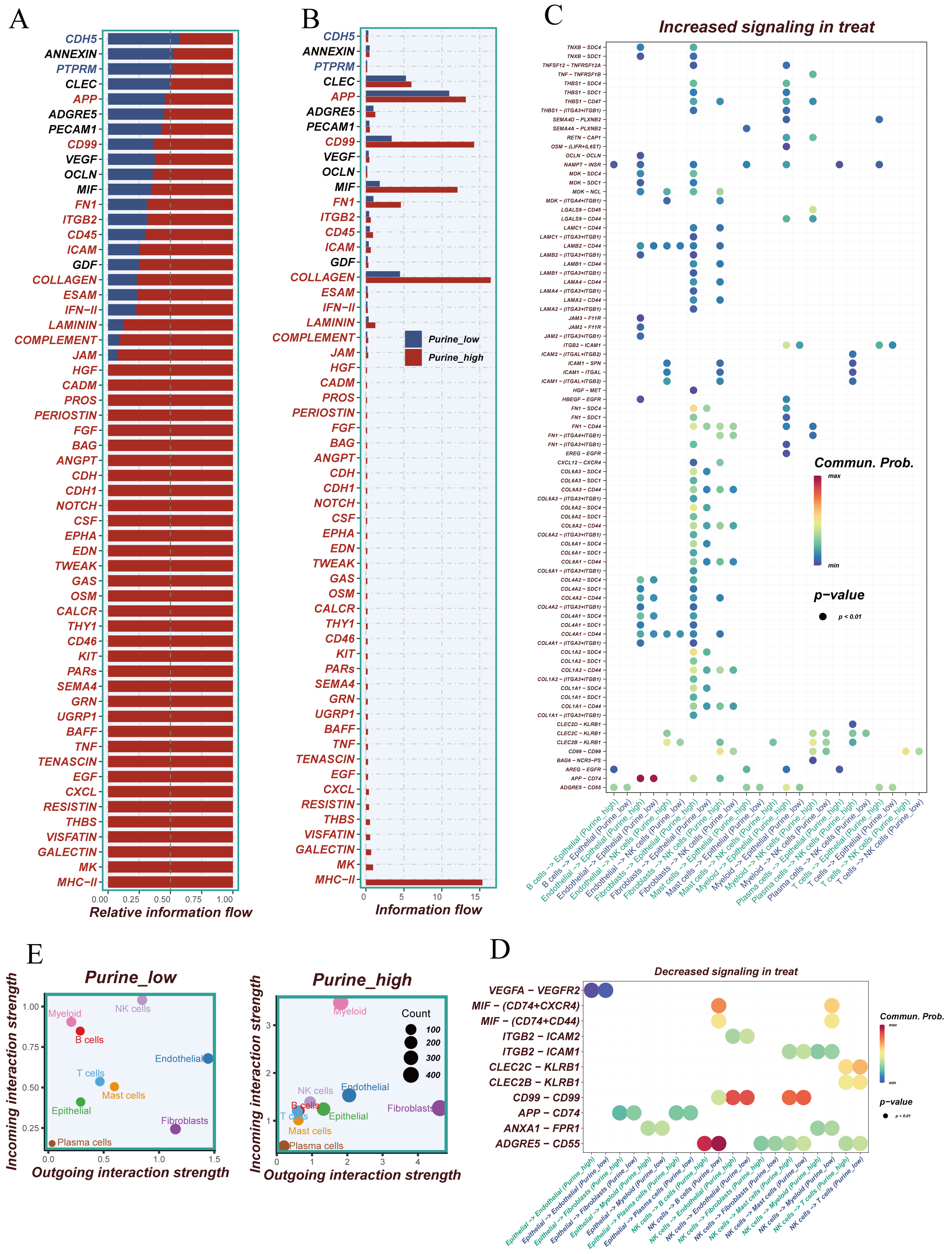

Supplement: Supplementary file 1 — Figure S1. Cell–cell interaction analysis. (A, B) Differential cell–cell interaction pathways between high and low PMS groups, illustrating the variance in intercellular communication mechanisms attributed to purine metabolism activity. (C, D) Variability in ligand‐receptor pairs facilitating cell–cell interactions across high and low PMS groups, highlighting the distinct molecular dialogue engaged by varying purine metabolic states. (E) Comparative analysis of communication strength among different cell types within high and low PMS groups, revealing alterations in intercellular signalling intensity associated with purine metabolic levels. [file JCMM-28-e18284-s002.tif]

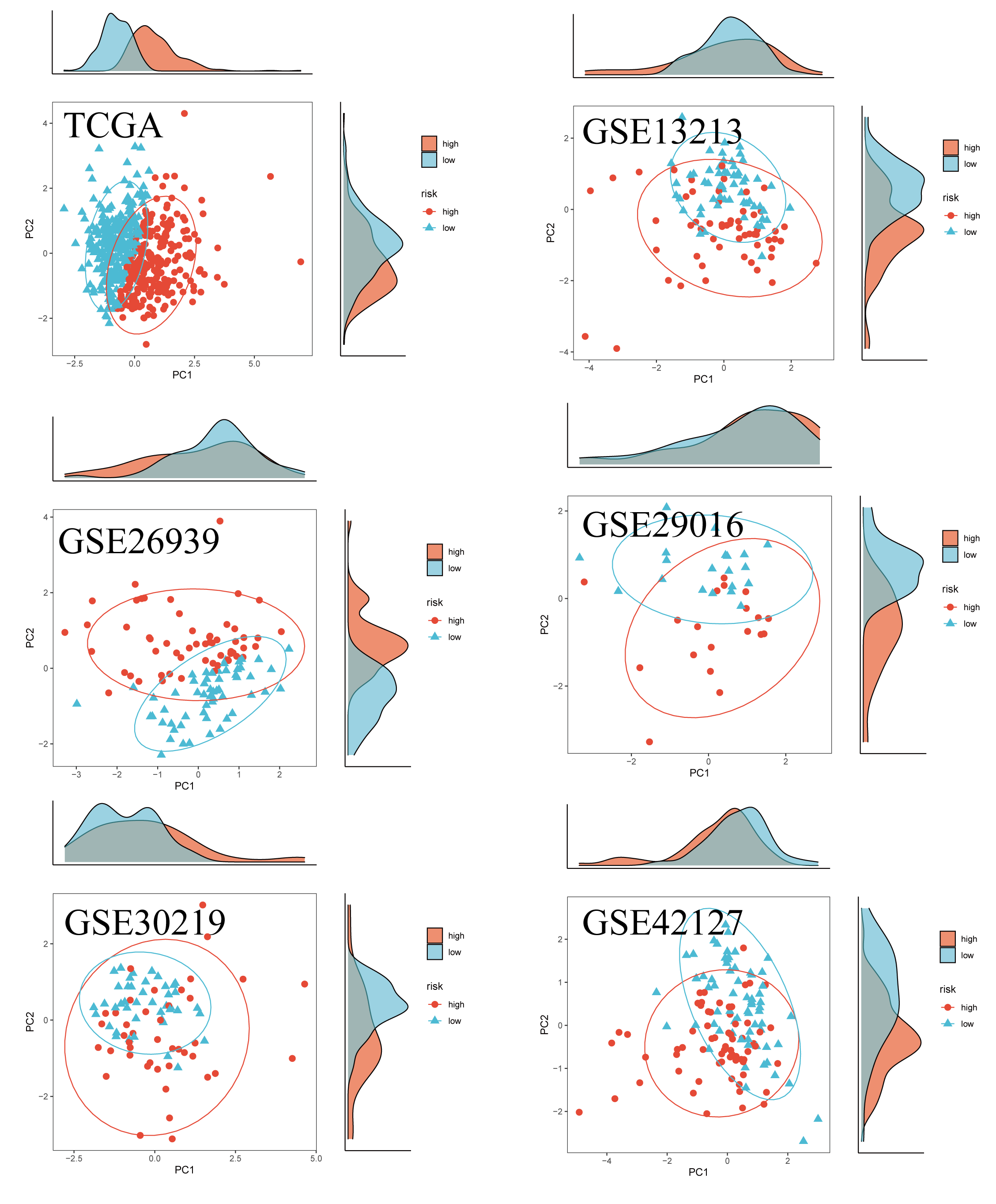

Supplement: Supplementary file 2 — Figure S2. PCA analysis shows that PAS scores can separate samples effectively in the TCGA, GSE13213, GSE26939, GSE29016, GSE30219 and GSE42127 datasets. [file JCMM-28-e18284-s004.tif]

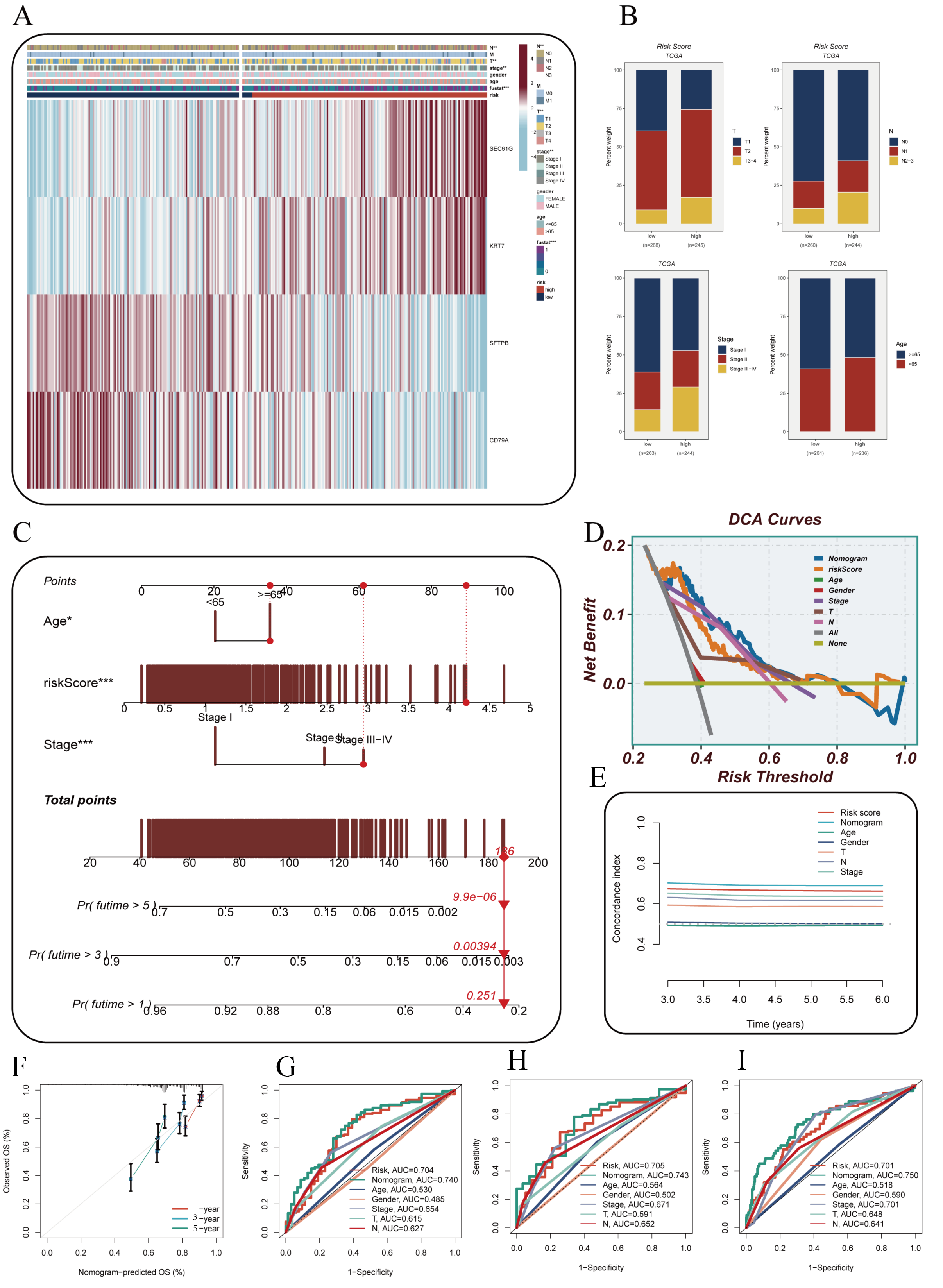

Supplement: Supplementary file 3 — Figure S3. Clinical correlation analysis. (A)Heatmap constructed by combining clinical features and model gene expression demonstrates the distribution of clinical features and model genes in two PAS groups. (B) Differences between high‐ and low‐PAS expression groups in terms of age, T, N and clinical stage. (C) Column line graph presenting a prognostic model for LUAD patients. (D) Decision curves. (E) C‐index curves. (F) Calibration curves. (G–I) ROC curves reflected the accuracy of the NOMO score in predicting prognosis. ***means p < 0.001; **means p < 0.01; ***means p < 0.05. [file JCMM-28-e18284-s003.tif]

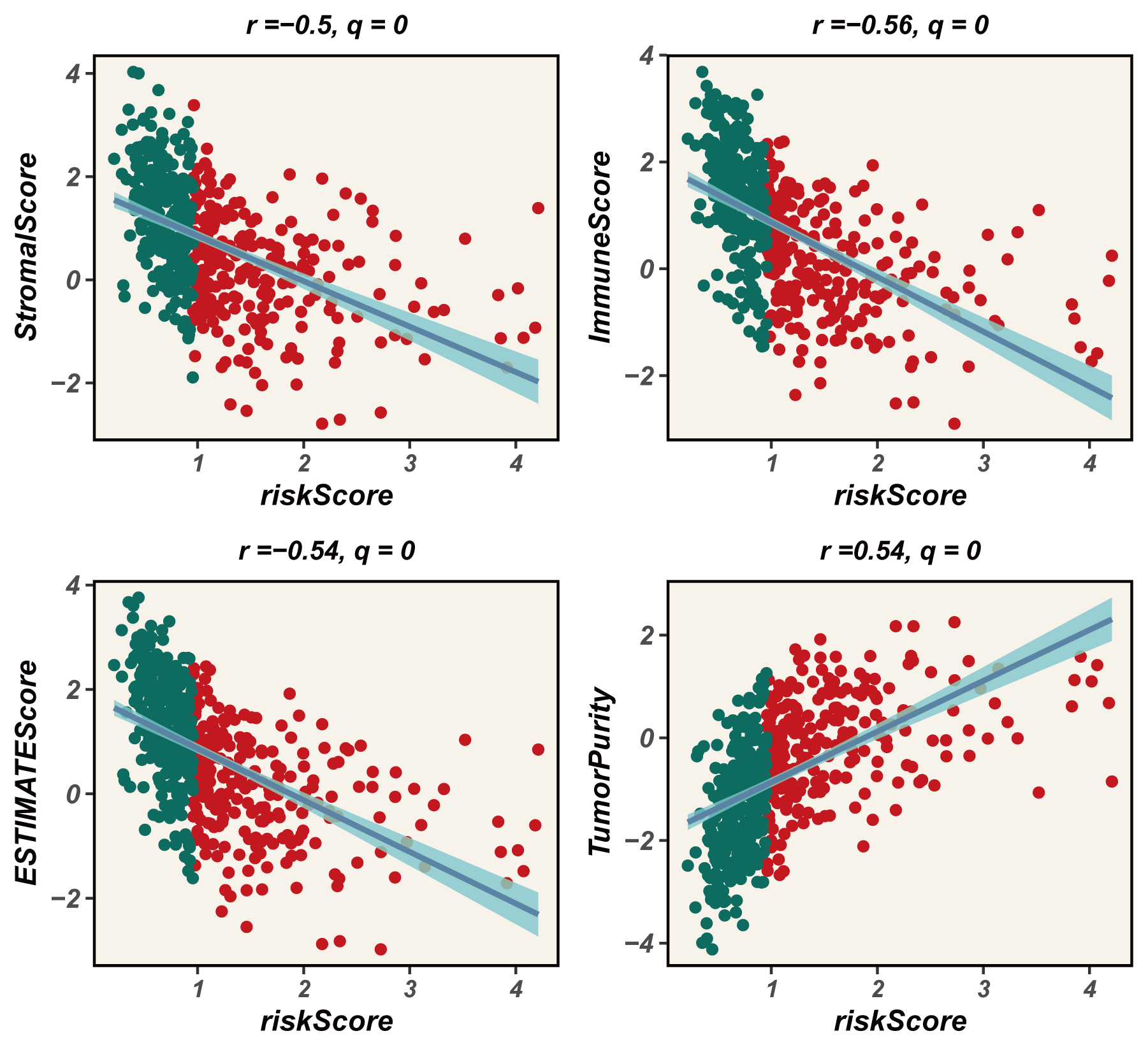

Supplement: Supplementary file 4 — Figure S4. The ESTIMATE R package was used to predict the correlation between PAS scores and immune scores, stromal scores, ESTIMATE scores and tumour purity. [file JCMM-28-e18284-s001.tif]
